# 

**Published:** 1877-07

**Authors:** 


### CONTENTS.

### COMMUNICATIONS.

The Principles Involved in the Treatment of Diseased Conditions. —*F. Peabody*.... 273
Mechanical Dentistry.—*C. B. Porter*.... 284
Osteo Sarcoma.—*F. Peabody, D. D. S*.... 287
Treatment of Exposed Pulps.—*F. C. Eddleman, D. D. S*.... 291
Plastic Dentistry.—*H. S. Chase, D. D. L*.... 294
Proceedings of Northern Ohio Dental Society .... 296

### SELECTIONS.

The use of Glycerine.... 307
Theory of the Action of Iodide of Potassium.... 308
A Case of Salivary Calculus.... 309
The Quanity of Drugs Used. .... 310

### EDITORIAL.

Microscopic .... 311
A Peculiar Tooth.... 311
An Obscure Case.... 312
American Dental Association.... 313
American Dental Convention.... 314
American Dental Association.... 316

